# Proline-Modified RWn Peptides: Enhanced Antifungal Efficacy and Synergy with Conventional Antibiotics for Combating Resistant Fungal Infections

**DOI:** 10.1021/acsomega.4c09054

**Published:** 2024-11-06

**Authors:** Nsoki Phambu, Anderson Sunda-Meya

**Affiliations:** † Department of Chemistry, Tennessee State University, Nashville, Tennessee 37209, United States; ‡ Department of Physics, Xavier University of Louisiana, New Orleans, Louisiana 70125, United States

## Abstract

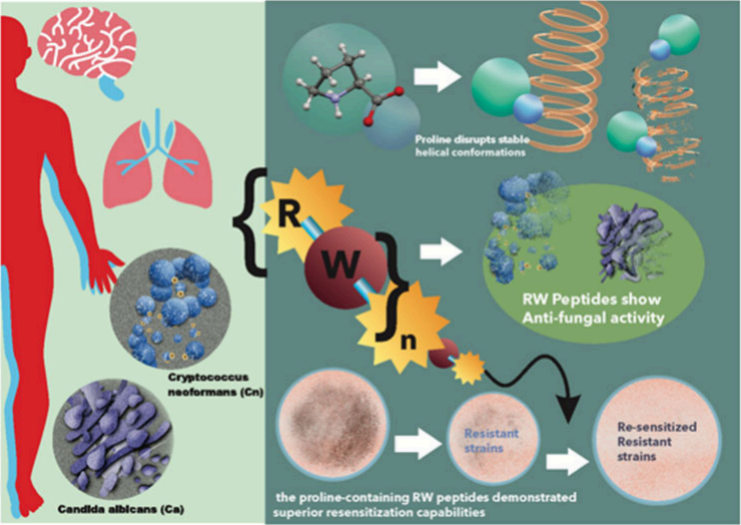

*Candida albicans* (Ca) and *Cryptococcus neoformans* (Cn) infections pose a growing threat due to rising antifungal resistance. This study explores a new class of antifungal agents, the RWn series (n = 4, 6, 8) peptides. These synthetic peptides were evaluated for their ability to inhibit Ca and Cn growth. All peptides except RW_4_ displayed antifungal activity, with RW_6_ exhibiting exceptional potency against Cn. Importantly, the incorporation of a proline residue significantly reduced cytotoxicity while maintaining antifungal activity against Cn for all RWnP peptides. Notably, RW_6_P demonstrated broad-spectrum activity against both Ca and Cn with low minimum inhibitory concentrations (MICs) and minimal toxicity. Furthermore, combining RW6P with trace amounts of traditional antibiotics (penicillin, vancomycin, and ampicillin) achieved synergistic effects, significantly reducing MICs against both fungi. These findings suggest that RWnP peptides, particularly RW6P, have promising potential as novel antifungal agents due to their high potency, broad-spectrum activity, and ability to resensitize fungi to existing antibiotics.

## Introduction

The escalating threat of antifungal resistance poses a formidable challenge to public health and imposes a substantial economic burden on healthcare systems globally.^1,2^ Fungal infections are a significant cause of morbidity and mortality, particularly among immunocompromised individuals.^1,3−5^ The diminished efficacy of antifungal therapy has exacerbated the clinical management of these infections.^6^ Annually, over 150 million severe fungal infections occur worldwide, resulting in approximately 1.7 million deaths, with these numbers steadily rising due to social and medical advancements facilitating the spread of these infections.^1^ In response to this escalating threat, the World Health Organization (WHO) released a list of 19 fungal strains classified as critical priority pathogens in 2022, representing the most severe threats to global health.^7^

Among these critical pathogens, *Candida albicans* and *Cryptococcus neoformans* are particularly concerning. *C. albicans* is the most prevalent human pathogenic fungus causing cutaneous infections, affecting 25% of the global population, with an annual incidence annually.^3^ As an opportunistic pathogen, *C. albicans* typically coinhabits the skin of healthy hosts but can be life-threatening to immunodeficient individuals. Alarmingly, recent research has shown that fungi have developed varying degrees of resistance to the four classes of antifungal drugs currently available.^3^*C. neoformans*, ranked as the highest priority fungal pathogen by the WHO’s Fungal Priority Pathogens List,^8^ poses a rising threat due to its ability to infect immunocompetent individuals and the emergence of antifungal-resistant variants. Cryptococcal meningitis, a fatal fungal central nervous system infection caused by *C. neoformans*, accounts for 19% of AIDS-related deaths annually.^8^

The pervasive use of azole fungicides in agriculture further exacerbates the issue of antifungal resistance, necessitating the development of next-generation antifungal agents with novel mechanisms of action, enhanced safety profiles, and different targets.^1^ In this context, antimicrobial peptides (AMPs) have garnered significant attention due to their safety, low residue, and low propensity for resistance development, with their unique antimicrobial mechanisms showing significant potential in combating antibiotic resistance.^9,10^ However, the high production costs and weak activity of AMPs limit their application.

Among the various AMP candidates, the RWn (n = 4,6,8) peptide series has emerged as a promising template for the synthesis of new antifungal agents.^5,11,12^ These peptides possess unique properties that confer high activity even at very short peptide lengths, owing to their richness in arginine (R) and tryptophan (W) residues. The W side-chain can interact with lipids in various ways, including hydrogen bonding, hydrophobic, π–π, cation-π, and anion-π interactions,^13−15^ while the R residues endow the peptides with cationic charges and hydrogen bonding properties necessary for interaction with the abundant anionic components of bacterial membranes.^12^ These peptides with repeating RW units are potent anti-infectives with great potential against pathogenic species such as bacteria, viruses, fungi, and parasites.^13,14^

Previous studies have evaluated the effect of chain length on the antimicrobial and antifungal activities of the RWn peptides.^5,12^ Liu et al.^12^ synthesized a series of peptides containing simple sequence repeats, (RW)n-NH_2_ (where n = 1, 2, 3, 4, or 5), and determined their antimicrobial and hemolytic activity. They found that the antimicrobial activity of the peptides increased with chain length, as did hemolysis of red blood cells. Within experimental error, longer peptides (n = 3, 4, or 5) showed similar values for the ratio of hemolytic activity to antibacterial activity or the hemolytic index. Gopal R et al.^5^ synthesized a similar series of peptides (where n = 2, 3, 4, or 5) and determined their antifungal activity, revealing that longer peptides displayed potent fungicidal activities against various agronomically important filamentous fungi.

Building upon these findings, Phambu et al.^11^ synthesized peptides containing (RW)n-NH_2_ units (where n = 4, 6, and 8) to study the effect of chain length on the structure and stability of the peptides using biophysical techniques. They concluded that RW6 was the most promising AMP, suggesting that repeating structures of (RW)n-NH_2_ promote the lateral assembly.

Against this backdrop, the present study aimed to (i) assess the antifungal effectiveness of the N-terminal peptides of the RWn series against the clinically relevant *Candida* strains *C. albicans* and *C. neoformans*, (ii) investigate whether chemical modification of the peptides by incorporating proline residues can enhance their safety and increase and/or broaden their antifungal activities, and (iii) explore whether the peptides of the RWn series can act as adjuvants to conventional antibiotics, potentially resensitizing these antibiotics against fungal strains.

Antibiotic-adjuvant combinatorial therapy has emerged as a promising orthogonal strategy to conventional antibiotic discovery,^16−23^ as the combination of antimicrobials can reduce dosages, attenuate negative side effects, and enhance the selectivity of compounds.^22^ Furthermore, AMPs can be used alongside antibiotics during treatment, typically leading to an increase in the lifetime of antibiotics, a reduction in the dosage of peptides needed for significant activity, and potentiation of the antibiotic effect.^19^

By conducting a comprehensive evaluation of the antifungal activities, cytotoxicity, hemolytic properties, and antibiotic adjuvant potential of the RWn peptides and their proline-containing derivatives, this study aimed to contribute to the development of effective antifungal strategies to combat the growing threat of antimicrobial resistance. The findings may pave the way for the design and optimization of novel antifungal agents based on the RWn peptide template, potentially addressing the urgent need for safe and effective therapeutics against fungal pathogens.

## Results and Discussion

### Synthesis and Characterization of RWn Peptides

Synthesis and characterization of the C-amidated peptides of the RWn (n = 4, 6, 8) series with and without proline residues were performed by GenScript Inc., New Jersey, USA.^24^ Proline residues are known to break helix conformation in peptides.^25−27^ GenScript carried out custom synthesis of the (RW)n peptides (n = 4, 6, 8) using high-efficiency solid-phase peptide synthesis. The purity (>96%), sequence, and molecular weight were assessed by RP-HPLC and ESI-MS, with experimentally measured molecular weights comparable to theoretical values (Table 1).^24,28−30^

**Table 1:** Molecular structures and physicochemical properties of reference peptides

Molecular formula or name	Code	Amino acid sequence	Observed MW (g/mol)	Net charge at pH ∼ 7	Length (Amino Acids)	Pathogen inhibited	Hemolytic Toxicity (<32 mg/mL)
(RW)4-NH2	RW4	RWRWRWRW	1386.62	+ 4	8	None	No
(RW)6-NH2	RW6	RWRWRWRWRWRW	2072.40	+ 6	12	*C. albicans*, *C. neoformans*	Yes
(RW)8-NH2	RW8	RWRWRWRWRWRWRWRW	2757.19	+ 8	16	*C. albicans*, *C. neoformans*	Yes
Indolicidin	INDO	ILPWKWPWWPWRR	1906.29	+ 3	13	None	No

The proline-containing RWn peptides were synthesized by incorporating one proline residue in the middle (RW4P, RW6P, RW8P) or between every two RW repeating units (RW6-2P) of the parent sequences. RW6 was chosen for proline modification as it was the most promising compound based on previous studies.^11^ The rationale for proline incorporation was to disrupt the helix structure known to confer rapid killing but also toxicity to antimicrobial peptides.^9,24^

### In Vitro Antifungal Activity and Toxicity

The antifungal activities were evaluated by determining the minimum inhibitory concentration (MIC) against *C. albicans* [ATCC 90028] and *C. neoformans* [ATCC 208821] that inhibits 99% fungal growth. Cytotoxicity and hemolytic activities were assessed against human embryonic kidney (HEK293) cells and human red blood cells, respectively. The antimicrobial screening was performed by The Community for Antimicrobial Drug Discovery (CO-ADD).^31^

### Antifungal Activity of RWn Peptides

The RWn peptides, consisting of repeated arginine (R) and tryptophan (W) units, were assessed for their antifungal activity against *C. albicans* and *C. neoformans*, two clinically relevant fungal strains that are major causes of infection in immunocompromised patients. These peptides were previously shown to possess potent antibacterial activity, and their antifungal properties were explored to evaluate their broader antimicrobial spectrum.^5,32−36^ All peptides exhibited excellent activities against both *Candida* strains, except RW4 which showed significant hemolytic toxicity (Table 2).

**Table 2:** Antifungal Activity and Toxicity of RWn Peptides

**Peptide**	***C. albicans*****MIC** (μg/mL)	***C. neoformans*****MIC** (μg/mL)	**HEK293 Toxicity** (μg/mL)	**Hemolytic Activity** (μg/mL)
RW4	>32	>32	>32	>32
RW6	8	≤0.25	>32	≤0.25
RW8	32	0.5	>32	0.452
Indolicidin	>32	>32	>32	>32

Consistent with previous observations on antibacterial activity,^5,12^ antifungal potency increased with chain length but was accompanied by higher hemolytic toxicity. RW4 showed poor antifungal activity, with MIC values greater than 32 μg/mL for both fungal species. It also exhibited significant hemolysis, making it unsuitable for further development without modification. With a MIC value of ≤0.25 μg/mL against *C. neoformans*, RW6 demonstrated the highest antifungal potency. However, its hemolytic activity at effective concentrations suggests that further optimization is needed to minimize the toxicity to human cells. RW8, the longest peptide in the series, exhibited moderate activity against *C. neoformans* (MIC = 0.5 μg/mL) but was significantly less effective against *C. albicans* (MIC = 32 μg/mL). The increase in chain length was correlated with higher hemolytic activity and potential toxicity to mammalian cells.

The results suggest that increasing the length of the peptide enhances antifungal activity but at the cost of increased toxicity, a pattern also observed in antibacterial studies. The high MIC values for *C. albicans* indicate that the RWn peptides, particularly RW4 and RW8, may be more selective for *C. neoformans*.

The superior antifungal activity of RW6 may be attributed to its balanced amphiphilicity and charge distribution, which allows for efficient disruption of the fungal membrane while maintaining a tolerable toxicity profile at low concentrations. However, the observed hemolysis underscores the need for structural modifications to improve the selectivity and reduce side effects.

Notably, the antifungal activities of the RWn peptides were superior to indolicidin, contradicting observations by Chan et al.^13^ who reported remarkable indolicidin activity against fungi. This discrepancy may arise from differences in peptide orientation (N-terminal vs C-terminal) and the specific fungal strains tested. To the best of our knowledge, this is the first study investigating the antifungal activity of N-terminal RWn peptides against these clinically relevant strains.

### Antifungal Activity of Proline-Containing RWn Peptides

The inclusion of proline residues into the RWn peptide series was explored as a method to enhance antifungal activity while reducing toxicity. Proline, known for its ability to disrupt stable secondary structures like alpha-helices, alters the structural dynamics of peptides, potentially improving their selectivity and reducing off-target effects on mammalian cells. The proline-containing RWn peptides exhibited superior antifungal activity compared to their proline-free counterparts, and notably, they demonstrated minimal cytotoxicity and hemolysis.

In this study, all proline-containing RWn peptides exhibited potent antifungal activity, particularly against *C. neoformans*. The MIC values ranged from 0.25 to 0.5 μg/mL, showing that even at low concentrations, these peptides could effectively inhibit fungal growth (Table 3). Notably, RW6P displayed remarkable broad-spectrum activity against both *C. albicans* and *C. neoformans*, with MIC values as low as 0.25 μg/mL, without observable toxicity in HEK293 or red blood cells.

**Table 3:** Antifungal Activity and Toxicity of Proline-Containing RWn Peptides

**Peptide**	***C. albicans*****MIC** (μg/mL)	***C. neoformans*****MIC** (μg/mL)	**HEK293 Toxicity**	**Hemolytic Activity**
RW4P	>32	≤0.25	>32	>32
RW6P	≤0.25	0.5	>32	>32
RW8P	>32	≤0.25	>32	>32
RW6–2P	≤0.25	≤0.25	>32	>32

Another noteworthy peptide is RW6-2P, where two proline residues were interspersed between RW units. This peptide exhibited remarkable antifungal activity (MIC = 0.25 μg/mL) against both fungal strains, again without any detectable toxicity. The addition of proline not only preserved or enhanced antimicrobial potency but also drastically reduced the hemolytic and cytotoxic properties that were observed in the longer proline-free peptides, such as RW8. This suggests that proline incorporation may modulate the amphiphilic properties of the peptides, facilitating the interaction with fungal membranes while sparing mammalian cell membranes.

These findings corroborate the hypothesis that proline disrupts stable helical conformations,^25−27^ modulating structural properties and enhancing antifungal selectivity while minimizing off-target effects.^9,37^ The reduced toxicity in the proline-containing peptides makes them particularly promising candidates for therapeutic development, as their use could mitigate the adverse side effects often associated with potent antimicrobial agents.

### RWn Peptides as Adjuvants to Conventional Antibiotics

Adjuvant therapies, which involve using a secondary agent to enhance the efficacy of a primary drug, have garnered significant interest as a strategy to combat antimicrobial resistance,^16−18,26^ although their toxicity profiles remain a challenge.^18^

In this study, RWn peptides were evaluated for their potential to enhance the antifungal activities of conventional antibiotics, specifically penicillin (PEN), vancomycin (VAN), and ampicillin (AMP), which were largely ineffective as stand-alone treatments against fungal strains.

When combined with trace amounts of RWn peptides, the activity of these antibiotics against *C. albicans* and *C. neoformans* was significantly enhanced. This improvement in efficacy is likely due to the ability of RWn peptides to disrupt fungal cell membranes, facilitating the entry and accumulation of antibiotics within the cells or through complementary actions on different cellular processes (Table 4).

**Table 4:** Antifungal Activity of RWn Peptides Combined with Conventional Antibiotics^a^

**Antibiotic**	**Peptide Adjuvant**	***C. albicans*****MIC** (μg/mL)	***C. neoformans*****MIC** (μg/mL)	**Toxicity (HEK293 and RBC)**
PEN	None	>32	>32	Significant
PEN	RW4	>32	>32	Toxic
PEN	RW6	>32	>32	Toxic
PEN	RW8	>32	>32	Toxic
PEN	RW6P	≤0.25	≤0.25	None
PEN	RW6–2P	≤0.25	≤0.25	None
VAN	None	>32	>32	Significant
VAN	RW4	32	≤0.25	Toxic
VAN	RW6	≤0.25	≤0.25	Minimal
VAN	RW6P	≤0.25	≤0.25	None
VAN	RW6–2P	≤0.25	≤0.25	None
AMP	None	>32	>32	Significant
AMP	RW4	2	>32	Toxic
AMP	RW6	32	≤0.25	Toxic
AMP	RW6P	≤0.25	≤0.25	None
AMP	RW6–2P	≤0.25	4	None

a 4.75 mg of selected antibiotics was mixed with 0.25 mg of peptide.

As a monotherapy, PEN showed no significant activity against either fungal species, with MIC values exceeding 32 μg/mL. However, when combined with RWn peptides, its antifungal efficacy was notably improved. RW6P, in particular, resensitized both *C. albicans* and *C. neoformans* to PEN, reducing the MIC to ≤0.25 μg/mL for both strains without associated toxicity. RW6-2P also showed strong adjuvant activity, particularly against *C. neoformans*.

VAN, typically ineffective against Gram-negative bacteria and certain fungal species due to its inability to penetrate outer membranes, demonstrated improved antifungal activity when paired with RWn peptides. RW4P and RW6P resensitized *C. neoformans* to VAN, reducing the MIC to ≤0.25 μg/mL without observable toxicity. The most effective combination was RW6P-van, which reduced the MIC to ≤0.25 μg/mL against both fungal strains.

Similarly to PEN and VAN, AMP exhibited poor antifungal activity as a monotherapy, with MIC values greater than 32 μg/mL. When combined with RWn peptides, AMP’s efficacy was significantly enhanced. RW6P-amp showed excellent resensitization of both *C. albicans* and *C. neoformans*, reducing the MIC value to ≤0.25 μg/mL without toxicity. RW8P-amp also exhibited strong adjuvant activity, though some toxicity was noted at higher concentrations.

The proline-containing peptides, particularly RW6P and RW6-2P, demonstrated the most robust adjuvant effects, enhancing the efficacy of all three antibiotics while maintaining a favorable safety profile. This is in contrast to the longer RW8 peptide, which showed higher toxicity when combined with antibiotics, likely due to its increased charge and length, which may enhance its membrane-disrupting capabilities but also increase its interaction with mammalian cells.

These findings, in alignment with previous reports of AMPs resensitizing bacteria to conventional antibiotics,^18^ suggest that RWn peptides, especially the proline-modified variants, hold significant potential as adjuvants in antifungal therapy. Their ability to resensitize resistant fungal strains to conventional antibiotics provides a promising strategy for overcoming antifungal resistance while minimizing the required antibiotic dosage and associated toxicity.

### Mechanism of Antifungal Activity and Synergy

The antifungal activity of RWn peptides is primarily attributed to their ability to disrupt microbial membranes through a combination of electrostatic interactions, hydrophobic forces, and membrane penetration. RWn peptides, composed of repeating arginine (R) and tryptophan (W) units, leverage the unique physicochemical properties of these amino acids to interact with and destabilize fungal membranes.^5,10,38,39^ Addition of proline to AMPs can also disrupt the alignment of amino acid side chains and this amphipathicity of AMPs.^40−42^

Tryptophan residues are critical for the interaction of RWn peptides with the microbial membranes. Tryptophan’s aromatic side chain can engage in hydrophobic interactions with lipid bilayers, penetrate deep into the membrane, and anchor the peptide. Additionally, tryptophan can form π–π, cation-π, and hydrogen-bonding interactions with membrane components, allowing the RWn peptides to disrupt the integrity of fungal membranes.^30,37^

For their part, arginine residues provide the cationic charge needed to target anionic fungal membrane components such as phospholipids and membrane-associated proteins. These strong electrostatic attractions between the positively charged arginine residues and the negatively charged membrane surface help initiate membrane disruption and facilitate the insertion of the peptide into the membrane.^38,43^ This facilitates the interaction of RWn peptides with fungal membranes, which results in the destabilization of membrane integrity, leading to increased permeability, ion leakage, and eventual cell lysis. The ability of RWn peptides to permeabilize fungal membranes is central to their mechanism of action. This permeabilization is often selective, targeting fungal cells more effectively than mammalian cells, which have different membrane compositions.^44,45^

### Synergy with Conventional Antibiotics

The combination of RWn peptides with conventional antibiotics, such as PEN, VAN, and AMP, is hypothesized to enhance their antifungal efficacy by multiple mechanisms. RWn peptides disrupt fungal cell membranes, allowing antibiotics that normally struggle with cell penetration to gain access to intracellular targets more effectively.^46,47^ For example, VAN, which cannot typically cross the outer membrane of Gram-negative bacteria or some fungal cells, becomes more effective when combined with RWn peptides. Complementary to that, while RWn peptides target fungal membranes, antibiotics such as AMP or PEN can inhibit essential intracellular processes, such as cell wall synthesis. This dual action—membrane disruption by the peptides and metabolic disruption by the antibiotics—leads to a synergistic effect, enhancing microbial killing.^44,48^

It has also been shown that the disruption of fungal membranes by RWn peptides may increase the intracellular concentration of antibiotics, allowing them to accumulate and act at higher local concentrations within the fungal cells.^44,47^

In addition, the inclusion of proline in the RWn peptide sequence further enhances the selectivity and minimizes toxicity. Proline introduces a kink in the peptide’s structure, preventing the formation of a stable α-helix, which alters the peptide’s ability to interact with cell membranes. This disruption of secondary structure can enhance membrane selectivity, enabling the peptide to target fungal cells more effectively while reducing the likelihood of damaging mammalian cells.^43,48^

Finally, the synergistic interactions between RWn peptides and conventional antibiotics (Table 5) may involve disruption of the fungal cell membrane by the peptides, facilitating antibiotic entry and accumulation within cells, thereby potentiating their efficacy.^17,19,46,49,50^ The combination of membrane-active peptides such as RWn with antibiotics that inhibit intracellular processes results in enhanced microbial killing. For instance, RW6P paired with VAN can reduce MIC values for resistant strains of *C. albicans* and *C. neoformans*, thereby allowing these antibiotics to work at lower, less toxic concentrations. This synergy is particularly beneficial in clinical settings where higher doses of antibiotics may result in side effects or toxicity.^51^

**Table 5:** Mechanism of Action and Synergistic Effects of RWn Peptides with Antibiotics

**Component/Property**	**Function/Role in Antifungal Activity**	**Synergy with Antibiotics**	**Selectivity (Fungal vs Mammalian Cells)**
Tryptophan Residues	Anchors peptide to membrane through hydrophobic & π–π interactions	Facilitates antibiotic entry by disrupting membrane	Strong interaction with fungal membranes, lower with mammalian
Arginine Residues	Provides positive charge, targets anionic membrane phospholipids	Increases membrane permeability, antibiotic accumulation	Essential for selective targeting of fungal cells
Membrane Permeabilization	Causes ion leakage, loss of membrane potential, cell lysis	Enhances antibiotic action by increasing cell access	Higher in fungal cells due to membrane composition differences
Proline Incorporation	Disrupts α-helix formation, increases membrane selectivity	Reduces peptide-induced toxicity, supports synergy	Reduces hemolysis and cytotoxicity while maintaining antifungal activity
Synergistic Interaction	Combines membrane disruption with intracellular inhibition	Antibiotics like VAN, PEN, and AMP are more effective	Synergy enables lower antibiotic dosages, reducing side effects

## Conclusion

This work clearly corroborates the potential of RWn peptides and especially those containing proline residues as highly active compounds against fungi that show low toxicity and selectivity for the latter organism. Selective killing of fungal cells by peptides without much damage to mammalian cells is enhanced through the disruption of the peptide secondary structures by the incorporation of proline. Among all the peptides, RW6P and RW6-2P were found to have the highest antifungal activity against both *C. albicans* and *C. neoformans* coupled with the least cytotoxicity to human cells.

A special focus should be made on the fact that RWn peptides can act as adjuvants to conventional antibiotics as well as other antifungal agents. RW6P and RW6-2P improved the effectiveness of PEN, VAN, and AMP by lowering the dose of antibiotics suggested and helping to prevent resistance development. These mechanisms of action, both membrane disruption and intracellular antibiotic activity, appear to be a sound strategy to combat antifungal resistance, an emerging issue in clinical practice.

Therefore, proline-modified RWn peptides can be concluded as a novel class of antimicrobial peptides and may be used as safe and novel therapeutic strategies against drug-resistant fungal infections. Their ability to supplement currently used antibiotics makes them more useful in practice since multidisciplinary approaches offer a better chance of controlling the chosen difficult to treat fungal pathogens. Further work should, however, aim at addressing the following: fine-tune the structure of the peptides by minimizing the toxicity values, conduct in vivo experiments, and evaluate RWn peptides against other species of fungi and multiple resistance strains.

## Methods

### Chemistry

#### Liquid Chromatography–Mass Spectrometry (LC/MS) Analysis of Synthetic Products

All synthesized molecules were characterized by using LC/MS to evaluate purity and identity.

#### Preparation of Samples for CO-ADD Submission

Peptides were prepared as dry materials for submission to the CO-ADD. Each sample consisted of 5 mg of pure compound, sufficient for primary screening, hit confirmation, cytotoxicity testing, and quality control (QC) if necessary. For antibiotic-AMP physical mixtures, the following nomenclature was used:

″PEN-RW6″ indicates a sample containing 95% PEN and 5% RW6 (w/w).

″RW6-PEN” indicates a sample containing 95% RW6 and 5% pen (w/w).

### Biology

#### Sample Preparation for Preliminary Screening

Samples received by CO-ADD were stored at −20 °C until use.

Compounds were dissolved in DMSO and water to achieve a final testing concentration of 32 or 20 μM.

Samples were prepared in 384-well, nonbinding surface plates for each bacterial/fungal strain.

All samples were tested in duplicate (n = 2).

The final DMSO concentration was maintained at a maximum of 1%.

All solution transfers were performed using liquid handling robots to ensure accuracy and reproducibility.

#### Preliminary Screening Assays

Antibacterial and antifungal screening assays were conducted following previously reported procedures. Detailed protocols, including growth media composition, incubation conditions, and readout methods, are provided in the Supporting Information.

#### Analysis of Preliminary Screening Results

1. Percentage growth inhibition was calculated for each treated well using the following formula: % Inhibition = [1 – (ODi – median(ODNegControl))/(median(ODPosControl) – median(ODNegControl))] × 100 Where: ODi = Optical density of the treated well; ODNegControl = Optical density of the negative control (media only); ODPosControl = Optical density of the positive control (untreated bacteria).
2. The significance of inhibition values was determined using modified Z-scores: Z-Score = 0.6745 × (ODi – median(OD))/MAD Where: MAD = Median Absolute Deviation.
3. Classification criteria:
4. Active: Inhibition >80% and Z-Score >2.5 for either replicate.
5. Partially active: Inhibition 50–80% and Z-Score >2.5 for either replicate.

#### Quality Control for Preliminary Screening

1. Positive control: Fluconazole was used as a fungal inhibitor standard for *C. albicans* and *C. neoformans*.
2. Z′-factor was calculated using positive and negative controls.
3. QC pass criteria:

- Z′-factor >0.4
- Antimicrobial standards showed full growth inhibition at their highest concentration and no inhibition at their lowest concentration.

#### Hit Confirmation Screening and MIC Determination

Compounds identified as partially active or active in the preliminary screen underwent hit confirmation screening and MIC determination.

#### Cytotoxicity Assay

Cytotoxicity assays were performed on active compounds to assess their selectivity toward microbial cells. The protocol for the cytotoxicity assay is described in the Supporting Information.^28^

#### Quality Control for Hit Confirmation

1. All screenings were performed in duplicate (n = 2) on different plates but from a single plating and screening event.
2. Quality control parameters for individual plates:

- Z′-Factor >0.4
- |median(ODPosControl) – median(ODNegControl)| > 3 × (MAD(ODPosControl) + MAD(ODNegControl))
- Standard antibiotic controls at concentrations above and below their MIC showed expected activity profiles.

## Supporting Information Available

Antimicrobial screening procedures and materials (PDF)

## Abbreviations

AMP: Ampicillin
AMPs: Antimicrobial Peptides
CO-ADD: The Community for Open Antimicrobial Drug Discovery
DMSO: Dimethyl Sulfoxide
ESI-MS: Electrospray Ionization Mass Spectrometry
MIC: Minimum Inhibitory Concentration
PEN: Penicillin
RP-HPLC: Reversed-phase High-Performance Liquid Chromatography
VAN: Vancomycin
