# Supplementary material for: Proline-Modified RWn Peptides: Enhanced Antifungal Efficacy and Synergy with Conventional Antibiotics for Combating Resistant Fungal Infections
Source: ACS Omega. 2024 Nov 6;9(46):46627–33. doi: 10.1021/acsomega.4c09054 (PMC11579929; doi:10.1021/acsomega.4c09054)
Supplement: Supplementary file 1 — ao4c09054_si_001.pdf [file ao4c09054_si_001.pdf]

Proline-Modified RWn Peptides: Enhanced Antifungal Efficacy and Synergy with Conventional Antibiotics for Combatting Resistant Fungal Infections

Nsoki Phambu[1] and Anderson Sunda Meya[2]*

[1]Department of Chemistry, Tennessee State University, Nashville, TN 37209, USA

[2]Department of Physics, Xavier University of Louisiana, New Orleans, LA 70125, USA

Primary Antimicrobial Screening

Bacterial and Fungal

Procedure and Materials

Content

1.0 Summary ... 3
1.1 Study ... 3
1.2 Assay Parameters ... 3
1.3 Comments ... 4
1.4 Publishing CO-ADD data ... 4
2.0 Methods ... 5
2.1 Sample preparation ... 5
2.2 Antimicrobial Assay ... 5
2.2.1 Procedure ... 5
2.2.2 Analysis ... 5
2.3 Antifungal Assay ... 5
2.3.1 Procedure ... 5
2.3.2 Analysis ... 6
2.4 Antibiotic standards preparation and Quality control ... 6
3.0 Materials ... 7
3.1 Assay materials ... 7
3.2 Standards ... 7
3.3 Microbial Strains ... 7
4.0 Controls ... 8
4.1 Antimicrobial susceptibility of tested strains ... 8
4.1.1 Antibiotic standards ... 8
4.1.2 Antifungal standard ... 9

1.0 Summary

1.1 Study

Primary antimicrobial screening study by whole cell growth inhibition assays, using the provided samples at a single concentration, in duplicate (n=2). The inhibition of growth is measured against 5 bacteria: *Escherichia coli*, *Klebsiella pneumoniae, Acinetobacter baumannii, Pseudomonas aeruginosa* and *Staphylococcus aureus*, and 2 fungi: *Candida albicans* and *Cryptococcus neoformans*.

1.2 Assay Parameters

TableS1. Assay parameters.

Test concentration		32 µg/mL or 20 µM<br>≤1% DMSO
QC		Duplicate (n=2)<br>Control MIC: Pass
Plates		Non-Binding Surface, 384 well plate
Media	***Bacteria***<br>***Fungi***	Cation-adjusted Mueller Hinton broth<br>Yeast Nitrogen Base
Read Out	***Bacteria***<br>***C. albicans***<br>***C. neoformans***	OD_{600}<br>OD_{530}<br>Resazurin $OD_{600-570}$

1.3 Comments

To confirm the inhibitory activity, the hit compound/s will be re-tested against the strains in a dose response assay to determine the minimum inhibitory concentration (MIC) of the compounds. Furthermore, to further evaluate the antimicrobial potential of the compounds they will be assayed against a mammalian cell line to determine general cell toxicity.

In order to continue with Hit Confirmation assays, CO-ADD requests (as per the standard T&C's) that chemical structures of the compound/s (both active and inactive) be supplied after receipt of the primary screening report. All structural information will be kept confidential and only used internally by CO-ADD for the purpose of evaluating novelty of the chemistry to choose compounds for further validation. No publication will result without your written consent.

If possible, please provide structures as **smiles, sdf/sd** or **cdx** files. If you do not have this means, images may also be accepted. Once we have received your structures, we will schedule the dose response assay of the active compound.

If you have not already provided structures to CO-ADD for your full compound set, please do so within a reasonable timeframe after receiving this report, so as not to delay Hit Confirmation.

2.0 Methods

2.1 Sample preparation

Samples were provided by the collaborator and stored frozen at -20 °C. Samples were prepared in DMSO and water to a final testing concentration of 32 µg/mL or 20 µM (unless otherwise indicated in the data sheet), in 384-well, non-binding surface plate (NBS) for each bacterial/fungal strain, and in duplicate (n=2), and keeping the final DMSO concentration to a maximum of 1% DMSO. All the sample-preparation where done using liquid handling robots.

Compounds that showed solubility issues during stock solution preparation are detailed in the data sheet.

2.2 Antimicrobial Assay

2.2.1 Procedure

All bacteria were cultured in Cation-adjusted Mueller Hinton broth (**CAMHB**) at 37 °C overnight. A sample of each culture was then diluted 40-fold in fresh broth and incubated at 37 °C for 1.5-3 h. The resultant mid-log phase cultures were diluted (CFU/mL measured by OD_{600}), then added to each well of the compound containing plates, giving a cell density of 5×10^5 CFU/mL and a total volume of 50 µL. All the plates were covered and incubated at 37 °C for 18 h without shaking.

2.2.2 Analysis

Inhibition of bacterial growth was determined measuring absorbance at 600 nm (OD_{600}), using a Tecan M1000 Pro monochromator plate reader. The percentage of growth inhibition was calculated for each well, using the negative control (media only) and positive control (bacteria without inhibitors) on the same plate as references. The significance of the inhibition values was determined by modified Z-scores, calculated using the median and MAD of the samples (no controls) on the same plate. Samples with inhibition value above 80% and Z-Score above 2.5 for either replicate (n=2 on different plates) were classed as actives. Samples with inhibition values between 50 - 80% and Z-Score above 2.5 for either replicate (n=2 on different plates) were classed as partial actives. Samples with inhibition values between 50 - 80% and Z-Score above 2.5 for either replicate (n=2 on different plates) were classed as partial actives.

2.3 Antifungal Assay

2.3.1 Procedure

Fungi strains were cultured for 3 days on Yeast Extract-Peptone Dextrose (**YPD**) agar at 30 °C. A yeast suspension of 1×10^6 to 5×10^6 CFU/mL (as determined by OD_{530}) was prepared from five colonies. The suspension was subsequently diluted and added to each well of the compound-containing plates giving a final cell density of fungi suspension of 2.5×10^3 CFU/mL and a total volume of 50 µL. All plates were covered and incubated at 35 °C for 24 h without shaking.

2.3.2 Analysis

Growth inhibition of *C. albicans* was determined measuring absorbance at 530 nm (OD_{530}), while the growth inhibition of *C. neoformans* was determined measuring the difference in absorbance between 600 and 570 nm ($OD_{600-570}$), after the addition of resazurin (0.001% final concentration) and incubation at 35 °C for additional 2 h. The absorbance was measured using a Biotek Synergy HTX plate reader. The percentage of growth inhibition was calculated for each well, using the negative control (media only) and positive control (fungi without inhibitors) on the same plate. The significance of the inhibition values was determined by modified Z-scores, calculated using the median and MAD of the samples (no controls) on the same plate. Samples with inhibition value above 80% and Z-Score above 2.5 for either replicate (n=2 on different plates) were classed as actives. Samples with inhibition values between 50 - 80% and Z-Score above 2.5 for either replicate (n=2 on different plates) were classed as partial actives.

2.4 Antibiotic standards preparation and Quality control

Colistin and Vancomycin were used as positive bacterial inhibitor standards for Gram-negative and Gram-positive bacteria, respectively. Fluconazole was used as a positive fungal inhibitor standard for *C. albicans and C. neoformans.*

The antibiotics were provided in 4 concentrations, with 2 above and 2 below its MIC value, and plated into the first 8 wells of column 23 of the 384-well NBS plates.

The quality control (QC) of the assays was determined by the antimicrobial controls and the Z'-factor (using positive and negative controls). Each plate was deemed to fulfil the quality criteria (pass QC), if the Z'-factor was above 0.4, and the antimicrobial standards showed full range of activity, with full growth inhibition at their highest concentration, and no growth inhibition at their lowest concentration.

3.0 Materials

3.1 Assay materials

Table S2. Assay materials.

Material	Code	Brand	Cat No.
Compound preparation plate [Polypropylene]	PP	Corning	3364
Assay Plates [Non-binding surface]	NBS 384w	Corning	3640
Growth media - bacteria	CAMHB	Bacto Laboratories	212322
Culture agar - fungi	YPD	Becton Dickinson	242720
Growth media - fungi	YNB	Becton Dickinson	233520
Resazurin		Sigma-Aldrich	R7017

3.2 Standards

TableS3. Standards

Sample Name	Sample ID	Full MW	Stock Conc. (mg/mL)	Solvent	Source
Colistin - Sulfate	MCC_000094:02	1400.63	10.0	DMSO	Sigma; C4461
Vancomycin - HCL	MCC_000095:02	1485.71	10.0	DMSO	Sigma; 861987
Fluconazole	MCC_008383:01	306.27	2.56	DMSO	Sigma; F8929

3.3 Microbial Strains

Table S4. Microbial Strains

ID	Batch	Organism	Strain	Description
FG_001	01	*Candida albicans*	ATCC 90028	CLSI reference
FG_002	01	*Cryptococcus neoformans*	ATCC 208821	H99 - Type strain

4.0 Controls

All antifungal controls displayed inhibitory values within the expected range. For further information please contact the CO-ADD team at support@co-add.org.

Table S5. Controls

Strain ID	***Species***	***Antibiotic***	***Pass/Fail***
FG_001:01	*C. albicans*	Fluconazole	Pass
FG_002:01	*C. neoformans* (H99)	Fluconazole	Pass

4.1 Antimicrobial susceptibility of tested strains

Values are the average of ≥ 6 independent biological replicates. All values are within the expected range as per CLSI guidelines.

4.1.1 Antifungal standard

Table S6. Antifungal standard

MIC determined by BMD method, YNB, Corning 3640 384 NBS plates		**FG_001:02**	**FG_002:02**
		Candida albicans	***Cryptococcus neoformans H99***
		CLSI reference	Type strain
		ATCC 90028	ATCC 208821
Compound	Compound Type	MIC (µg/mL)	
Fluconazole	Antifungal	**0.125**	**8**
